# Editorial: Maternal-Perinatal Risk and Children-Adolescent Health

**DOI:** 10.3389/fpubh.2021.744448

**Published:** 2022-01-06

**Authors:** Julian Alberto Herrera-Murgueitio, Patricio Lopez-Jaramillo, Kristine Koski, Adalberto Sanchez, Juan Pablo Herrera-Escobar

**Affiliations:** ^1^School of Medicine, Universidad del Valle, Cali, Colombia; ^2^Medical School, Masira Research Institute, Universidad de Santander, Bucaramanga, Colombia; ^3^School of Human Nutrition, McGill University, Sainte-Anne-de-Bellevue, QC, Canada; ^4^School of Basic Sciences, Violence and Adolescent Research Institute (CISALVA), Universidad del Valle, Cali, Colombia; ^5^Center for Surgery and Public Health, Harvard Medical School, Brigham and Women's Hospital, Boston, MA, United States

**Keywords:** maternal-perinatal risk, children health, adolescent health, social stress, inflammatory response

Environmental and behavioral factors during pregnancy and perinatal periods have been associated with the future development of health disorders, particularly with non-communicable chronic diseases. Maternal infections, nutrient deficiencies, and social stresses have been described as factors that are highly relevant in low and medium-income countries (1). The present issue of Frontiers has been dedicated to explore the topic Maternal-Perinatal Risk and Children-Adolescent Health, which gave us the opportunity to examine this topic with different approaches. In addition, the results of the articles published in the present issue open doors for future research and interventions in this important topic.

The paper of Chang et al. analyzed a big database from Taiwan, one of the leading countries with outstanding assistance in reproductive technology and modern medical care, and reported a higher rate of low birth and preterm birth as compared to those in the natural pregnancy group. This suggested that further evaluation and tighter regulations may be needed with regards to the use of this technology. In this context, Mei et al. described that in Traditional Chinese Medicine, the herbal recipe *Bushen Yutai* was able to increase and produce higher quality embryos, both of which have important implications to *in vitro* reproduction. The results of González-Fernández et al. showed that maternal stress in women from a conflict zone was associated with biomarkers of maternal-fetal well-being, highlighting the importance of assessing and addressing stress in pregnant women from vulnerable communities. Torres-Muñoz et al. described, in low-income pregnant women, that the assessment of biological and psychosocial factors and risk behaviors was important to determine the risk of developing perinatal asphyxia. Moreover, the same group showed that late premature infants were products of mothers with morbidity (specifically hypertensive disorders), had greater odds to be born by cesarean section, had a higher probability of developing morbidity, and more often, needed early obstetric intervention and required further resuscitation. Moreover, Torres-Salazar et al. found in women with preeclampsia differential methylation in the glucocorticoid receptor (GR) NR3C1 and its co-chaperone HSP90AA1. They suggested a possible regulatory role of this receptor in the response to stress during pregnancy and also a physio-pathological role in preeclampsia. In order to investigate factors impacting the risk of child maltreatment, Kawaguchi et al. revealed that child maltreatment was associated with maternal younger age, fathers much older than mothers, and single mothers. The evidence suggested a causal relationship between non-sexual child maltreatment and a range of mental disorders, drug use, suicide attempts, sexually-transmitted infections, and risky sexual behavior during childhood and into adulthood. All forms of child maltreatment should be considered as important risk factors to illness with a sizeable impact on major contributors to the burden of disease in all countries (2).

Obesity and overweight are linked with gestational diabetes and preeclampsia, the most important causes of maternal and perinatal morbidity and mortality in developing countries. Liu et al. presented the results of a meta-analysis demonstrating that pre-pregnancy maternal overweight or obesity and also high or low gestational weight gain rendered their offspring susceptible to an increased risk of asthma and wheeze during childhood. These results demonstrated the importance of appropriate antenatal health that includes maternal nutritional evaluation. The importance of maternal nutrition and child health is highlighted by the results of Yu et al., who reported that vitamin D supplementation during pregnancy could effectively reduce the risk of its deficiency in neonates. Adequate nutrition during pregnancy is important for fetal growth and for better maternal immunity response. Martinez-Lopez et al. demonstrated that the eating behavior of a family is determined by the meaning that the caretaker gives to food, by the act of eating in the domestic environment, and by beliefs and perceptions around those concepts. The common domains are well-being, health maintenance, coexistence, and security. Pregnancy has adverse effects on the prognosis of cervical cancer. Li et al. described the epidemiological characteristics, clinical features, clinical management, and maternal and infant outcomes of patients with cervical cancer during pregnancy. They found that an early diagnosis improved the prognosis of cervical cancer during pregnancy.

In a second article from Gonzalez-Fernandez et al., the association of maternal infections, nutrient deficiencies, and inflammation (MINDI) with maternal blood pressure and symphysis-fundal-height were explored, showing that mean arterial blood pressure and pulse pressure were useful tools to detect women at risk of adverse pregnancy outcomes in settings that have limited access to technology. In this context, Samwel et al. reported that bacterial vaginosis (BV) predisposed women and their babies to an increased frequency of illness. They showed a higher frequency of gastrointestinal morbidity among BV-HIV-1-exposed infants, observing that at 6 and 12 months, infants from mothers with BV had higher odds of bloody stool dehydration, vomiting, and mouth ulcers.

The impact of early life stress on postnatal brain development has been reviewed by González-Acosta et al. The understanding of this relationship has allowed for a better formulation and implementation of preventive measures, as well as the reorientation of research targets to improve health outcomes in all populations. Wang et al. observed a significant geographic disparity and an association between anemia and stunting among specific groups of school-aged children in China and thereby suggested that eliminating the geographic disparity and ameliorating stunting might contribute to the improvement of anemia, especially in adolescent girls and in groups with serious anemia burden. In a prospective birth cohort study in Shanghai, Li et al. described the impact of early life pet ownership on allergic sensitization and atopic dermatitis showing that half of the preschool children had positive allergen sensitization even in non-atopic children. Although early life exposure to dogs may increase the risk of allergic sensitization, it significantly decreased the risk of atopic dermatitis.

Early life stress can be caused by acute or chronic exposure to childhood events, such as emotional, physical, sexual abuse, and neglect. Early life stress is associated with subsequent alterations in physical and mental health, which can extend into adolescence, adulthood, and older age. The evidence suggests a causal relationship between non-sexual child maltreatment and a range of mental disorders, drug use, suicide attempts, sexually-transmitted infections, and risky sexual behavior during childhood and adulthood (2, 3) as discussed by Gonzalez et al. in this special issue.

Biomedical and psychosocial risk factors during pregnancy increase inflammatory response involved in the development of maternal diseases affecting fetal growth (3). Low birth weight is a marker of poor fetal growth and nutrition, and it is linked to coronary artery disease, hypertension, obesity, and insulin resistance (4). Early assessment and intervention of biomedical and psychosocial risk can prevent perinatal mortality, morbidity, and prevent child maltreatment (5). This special issue reported new potential links between maternal-perinatal risk environmental factors and children and adolescent health. The heterogeneity of articles published in this special topic highlights that maternal, perinatal, and children and adolescent health need to be approached in a multidisciplinary fashion.

## Author Contributions

JH-M, PL-J, KK, and AS were the associate editors. JH-E was editorial author. All authors contributed to the article and approved the submitted version.

## Conflict of Interest

The authors declare that the research was conducted in the absence of any commercial or financial relationships that could be construed as a potential conflict of interest.

## Publisher's Note

All claims expressed in this article are solely those of the authors and do not necessarily represent those of their affiliated organizations, or those of the publisher, the editors and the reviewers. Any product that may be evaluated in this article, or claim that may be made by its manufacturer, is not guaranteed or endorsed by the publisher.
